# Association of the Triglyceride–Glucose Index with Contrast-Associated Acute Kidney Injury in Patients with Atherosclerotic Renal Artery Stenosis

**DOI:** 10.3390/jcdd13090447

**Published:** 2026-09-08

**Authors:** Ece Celebi Coskun, Mahmut Kapsiz

**Affiliations:** Department of Cardiology, University of Health Sciences Bursa Yuksek Ihtisas Training and Research Hospital, 16310 Bursa, Türkiye; mahmutapsiz@gmail.com

**Keywords:** atherosclerotic renal artery stenosis, contrastassociated acute kidney injury, lipoprotein combined index, triglyceride–glucose index

## Abstract

Contrast-associated acute kidney injury (CA-AKI) is an important complication of renal angiography in patients with atherosclerotic renal artery stenosis (RAS). We investigated the associations of the triglyceride–glucose (TyG) index, atherogenic coefficient (AC) and lipoprotein combined index (LCI) with CA-AKI in this population. This retrospective single-center study included 147 consecutive patients with atherosclerotic RAS undergoing renal angiography with or without renal artery intervention. CA-AKI was defined using a creatinine-based definition based on serum creatinine changes within 48–72 h after contrast exposure. Continuous variables were retained in their continuous form in the primary logistic regression analyses, and separate multivariable models were constructed for the TyG index, AC, and LCI, each adjusted for chronic kidney disease (CKD), left ventricular ejection fraction (LVEF), and contrast volume. Firth penalized logistic regression was performed as a sensitivity analysis. CA-AKI occurred in 22 patients (15.0%). In the adjusted model, a higher TyG index was associated with increased odds of CA-AKI (OR 4.263 per 1-unit increase, 95% CI 1.120–16.228; *p* = 0.034), whereas AC (OR 1.581, 95% CI 0.891–2.807; *p* = 0.118) and LCI (OR 1.047 per 10,000-unit increase, 95% CI 0.966–1.136; *p* = 0.264) were not significantly associated with CA-AKI. The association of the TyG index with CA-AKI remained significant in Firth penalized logistic regression (OR 3.48, 95% CI 1.17–14.33; *p* = 0.020). Higher contrast volume and lower LVEF were consistently associated with CA-AKI across the multivariable models. These findings suggest that the TyG index is associated with susceptibility to CA-AKI in patients with atherosclerotic RAS, although prospective validation is required before clinical application.

## 1. Introduction

Atherosclerotic renal artery stenosis (RAS) is a common manifestation of systemic atherosclerosis and is associated with resistant hypertension, progressive renal dysfunction, ischemic nephropathy, recurrent flash pulmonary edema, and an increased risk of cardiovascular events. A recent meta-analysis including nine randomized controlled trials confirmed the findings of landmark randomized studies, demonstrating no significant advantage of routine renal artery revascularization over optimal medical therapy with respect to cardiovascular or renal outcomes [1,2,3]. Accordingly, current guidelines recommend renal artery intervention for carefully selected patients with hemodynamically significant stenosis and high-risk clinical features, such as resistant hypertension, rapidly progressive renal dysfunction, recurrent flash pulmonary edema, or otherwise unexplained heart failure [4]. Nevertheless, because renal artery intervention continues to be performed in these patients, minimizing procedure-related complications, particularly contrast-associated acute kidney injury (CA-AKI), remains of considerable clinical importance.

CA-AKI is one of the most frequent and clinically relevant complications following procedures requiring iodinated contrast media. It is associated with prolonged hospitalization, progression of chronic kidney disease, increased cardiovascular morbidity, and higher mortality [5,6]. Despite established clinical risk factors, identification of patients at increased risk of CA-AKI remains challenging, highlighting the need for simple and readily available biomarkers that may complement conventional preprocedural risk assessment.

Increasing evidence suggests that metabolic abnormalities, including insulin resistance, atherogenic dyslipidemia, oxidative stress, endothelial dysfunction, and chronic low-grade inflammation, play pivotal roles in the pathogenesis of CA-AKI. These interconnected mechanisms contribute to renal microvascular dysfunction, impaired renal perfusion, and enhanced susceptibility to renal injury following contrast exposure [7]. Consequently, several laboratory-derived metabolic biomarkers have recently emerged as potential markers for identifying patients with increased susceptibility to CA-AKI before contrast exposure.

Among the metabolic biomarkers proposed for cardiovascular risk assessment, the triglyceride–glucose (TyG) index, atherogenic coefficient (AC), and lipoprotein combined index (LCI) have attracted increasing attention because they integrate different aspects of metabolic dysfunction, including insulin resistance, oxidative stress, inflammation, and atherogenic dyslipidemia. The TyG index, a well-established surrogate marker of insulin resistance, has been shown to predict CA-AKI in patients undergoing coronary angiography and percutaneous coronary intervention [8,9]. AC, calculated as the ratio of non- high-density lipoprotein cholesterol (non-HDL-C) to high-density lipoprotein cholesterol (HDL-C), is a simple lipid-derived index reflecting the balance between atherogenic and anti-atherogenic cholesterol fractions and has been investigated as a marker of cardiovascular risk [10]. LCI, a composite lipid index integrating total cholesterol, triglycerides, low-density lipoprotein cholesterol (LDL-C), and high-density lipoprotein cholesterol (HDL-C), provides a comprehensive measure of overall atherogenic burden and has demonstrated prognostic value in patients with cardiovascular disease [10,11]. However, despite their established associations with cardiovascular risk, the associations of the TyG index, AC, and LCI with CA-AKI among patients with atherosclerotic renal artery stenosis undergoing renal angiography have not yet been investigated.

Therefore, the present study aimed to evaluate the associations of the TyG index, AC, and LCI with the development of CA-AKI in patients with atherosclerotic renal artery stenosis undergoing renal angiography with or without renal artery intervention. We also sought to determine whether these associations persisted after adjustment for clinically relevant renal, cardiac, and procedural factors.

## 2. Materials and Methods

### 2.1. Study Design and Population

This retrospective, observational, single-center study included consecutive patients with atherosclerotic RAS who underwent renal angiography with or without renal artery intervention at Bursa Yuksek Ihtisas Training and Research Hospital between 6 September 2022 and 23 June 2026. Patients younger than 18 years, those with non-atherosclerotic RAS (e.g., fibromuscular dysplasia or vasculitis), end-stage kidney disease requiring chronic dialysis, previous kidney transplantation, active malignancy, active infection or sepsis, advanced liver disease, hematological disorders potentially affecting laboratory parameters, missing laboratory data required for calculation of the investigated biomarkers, or unavailable post-procedural serum creatinine measurements were excluded. The study flow diagram is shown in Supplementary Figure S1.

RAS was diagnosed by conventional renal angiography. The severity of stenosis was visually assessed by experienced interventional cardiologists. Luminal diameter narrowing of ≥70% was defined as critical RAS, whereas stenosis of <70% was classified as non-critical. Lesion location was categorized as ostial or distal, and the target vessel was classified as the right, left, or bilateral renal artery. The decision to perform renal artery intervention was made at the discretion of the treating interventional cardiologist according to contemporary guideline recommendations and individual clinical indications.

The study was approved by the Ethics Committee of the University of Health Sciences Bursa Yuksek Ihtisas Training and Research Hospital (Approval No. 2024-TBEK-2026/08-11, Approval Date: 5 August 2026) and was conducted in accordance with the principles of the Declaration of Helsinki. Owing to the retrospective nature of the study, the requirement for informed consent was waived.

### 2.2. Clinical, Laboratory, Echocardiographic, and Angiographic Data

Baseline demographic characteristics, clinical variables, laboratory findings, echocardiographic measurements, renal angiographic characteristics, and procedural data were retrospectively retrieved from the hospital electronic medical record system. Demographic and clinical variables included age, sex, severity of RAS (critical or non-critical), lesion location (ostial or distal), target renal artery (right, left, or bilateral), hypertension, diabetes mellitus, coronary artery disease, cerebrovascular disease, chronic kidney disease, and antihypertensive treatment regimen.

Baseline laboratory parameters obtained before renal angiography included hemoglobin, neutrophil, lymphocyte, monocyte, and platelet counts, fasting plasma glucose, glycated hemoglobin (HbA1c), triglycerides, total cholesterol, LDL-C, HDL-C, serum creatinine, estimated glomerular filtration rate (eGFR) calculated using the Modification of Diet in Renal Disease (MDRD) equation, serum uric acid, and serum albumin. Left ventricular ejection fraction (LVEF) was assessed before the procedure by transthoracic echocardiography using the modified biplane Simpson method in accordance with current echocardiographic recommendations. Procedural data included contrast volume and procedure type, with patients classified as undergoing diagnostic renal angiography alone or renal artery intervention. A non-ionic, low-osmolar iodinated contrast medium (iohexol, 350 mg iodine/mL) was used in all patients. Although different commercially available formulations of iohexol were used during the study period, the active contrast agent and iodine concentration were consistent, and the contrast protocol otherwise remained unchanged throughout the study period.

### 2.3. Calculation of Metabolic Biomarkers

The investigated metabolic biomarkers were calculated using baseline laboratory measurements obtained before renal angiography according to previously published formulas. TyG index was calculated as ln [fasting triglycerides (mg/dL) × fasting plasma glucose (mg/dL)/2]. The atherogenic coefficient (AC) was calculated as (total cholesterol -HDL-C)/HDL-C, whereas the LCI was calculated as (total cholesterol × triglycerides × LDL-C)/HDL-C.

### 2.4. Study Outcomes

CA-AKI was defined using the serum creatinine-based definition as an increase in serum creatinine of ≥0.3 mg/dL (≥26.5 μmol/L) or ≥50% from baseline within 48–72 h after contrast exposure. Urine-output criteria were not assessed because these data were not consistently available in the retrospective medical records [12].

The secondary outcome was a composite clinical endpoint comprising all-cause mortality, myocardial infarction, stroke, hospitalization for heart failure, and renal artery restenosis requiring repeat revascularization during follow-up.

### 2.5. Statistical Analysis

The distribution of continuous variables was assessed using the Kolmogorov–Smirnov test and visual inspection of histograms and Q-Q plots. Because the continuous variables were not normally distributed, they are presented as medians and interquartile ranges (IQRs). Categorical variables are presented as frequencies and percentages. Comparisons between patients with and without CA-AKI were performed using the Mann–Whitney U test for continuous variables and the Pearson chi-square test or Fisher’s exact test, as appropriate, for categorical variables.

Univariable logistic regression analyses were performed to evaluate factors associated with CA-AKI. Continuous variables were entered into the logistic regression analyses in their continuous form to avoid information loss and potential bias associated with data-derived dichotomization. Odds ratios (ORs) and corresponding 95% confidence intervals (CIs) were calculated per one-unit increase in each continuous variable. Because of its large numerical scale, LCI was rescaled and expressed per 10,000-unit increase.

Given the limited number of CA-AKI events, multivariable model complexity was deliberately restricted. CKD, LVEF, and contrast volume were included as clinically relevant adjustment variables representing baseline renal vulnerability, cardiac function, and procedural contrast exposure, respectively, rather than selecting covariates solely according to statistical significance in univariable analyses. CKD was retained as the clinically selected measure of baseline renal vulnerability. Given the limited number of CA-AKI events and the overlapping information conveyed by CKD and continuous eGFR, both variables were not entered simultaneously to avoid unnecessary model complexity and redundancy. Separate multivariable logistic regression models were constructed for the investigated metabolic indices. Model A included CKD, LVEF, contrast volume, and the TyG index; Model B included CKD, LVEF, contrast volume, and AC; and Model C included CKD, LVEF, contrast volume, and LCI.

Given that the TyG index and LCI both incorporate triglycerides in their formulas, Spearman rank correlation analysis was performed to assess the correlations among the TyG index, LCI, and triglyceride levels and to characterize the degree of shared metabolic information among these variables. Multicollinearity among covariates included in each multivariable model was assessed using tolerance and variance inflation factor (VIF) values. The linearity-in-the-logit assumption for continuous predictors was assessed using Box–Tidwell interaction terms.

Because of the limited number of CA-AKI events, Firth penalized logistic regression was additionally performed as a sensitivity analysis to reduce potential small-sample bias and assess the robustness of the conventional logistic regression estimates. The same covariate structure was retained in the corresponding penalized models. Firth penalized logistic regression analyses were performed using MedStat, a web-based statistical analysis platform, with complete-case analysis. Models A and B included 147 patients, whereas Model C included 143 patients with complete LCI data.

Receiver operating characteristic (ROC) curve analyses were performed as exploratory analyses to characterize the discriminatory ability of selected continuous variables and to identify data-derived thresholds. Because these thresholds were derived and evaluated within the same study cohort, they were not used to dichotomize variables in the primary logistic regression analyses and should therefore be regarded as exploratory.

All other statistical analyses were performed using IBM SPSS Statistics for Windows, version 25.0 (IBM Corp., Armonk, NY, USA). A two-sided *p* value < 0.05 was considered statistically significant.

## 3. Results

The baseline demographic, clinical, and laboratory characteristics of the study population according to the development of CA-AKI are presented in Table 1. A total of 147 patients were included, of whom 22 (15.0%) developed CA-AKI following renal angiography. There were no significant differences between the groups with respect to age, sex, lesion location, target renal artery, hypertension, coronary artery disease, cerebrovascular disease, or antihypertensive treatment regimen (all *p* > 0.05). Although diabetes mellitus was more frequent among patients who developed CA-AKI, the difference did not reach statistical significance (45.5% vs. 26.4%, *p* = 0.070). Pre-existing chronic kidney disease (CKD) was significantly more prevalent in the CA-AKI group (68.2% vs. 23.2%, *p* < 0.001). In addition, the distribution of renal artery stenosis severity differed significantly between the groups (*p* = 0.002). Renal artery intervention was performed in 31 patients (21.1%) and did not differ significantly according to CA-AKI status (*p* = 0.085). The median implanted stent diameter was 6.0 mm (IQR, 5.0–7.0 mm), whereas the median stent length was 18.0 mm (IQR, 15.0–19.0 mm). In-hospital dialysis was required in two patients, both of whom developed CA-AKI.

Patients who developed CA-AKI also received significantly higher contrast volumes than those without CA-AKI (70.00 [65.00–86.00] mL vs. 60.00 [55.00–60.00] mL, *p* < 0.001). Contrast volume was significantly higher in patients undergoing renal artery intervention than in those undergoing renal angiography alone (70 [60–80] vs. 60 [55–60] mL, *p* < 0.001). Patients who developed CA-AKI had significantly lower LVEF, hemoglobin, lymphocyte count, HDL-C, serum albumin levels, and eGFR levels, whereas glucose, triglyceride, serum creatinine, and uric acid levels were significantly higher than those in patients without CA-AKI (all *p* < 0.01). The distribution of eGFR categories (≥60, 30–59, and <30 mL/min/1.73 m^2^) also differed significantly according to CA-AKI status (Fisher’s exact *p* = 0.001). Among the investigated metabolic indices, the TyG index (9.60 [9.19–10.09] vs. 9.02 [8.59–9.55], *p* = 0.001), atherogenic coefficient (4.32 [3.52–5.51] vs. 3.64 [2.46–4.53], *p* = 0.018), and lipoprotein combined index (111,022.96 [83,447.25–178,419.05] vs. 69,696.00 [32,868.77–165,605.83], *p* = 0.045) were significantly higher in patients with CA-AKI. No significant differences were observed in neutrophil count, monocyte count, platelet count, HbA1c, LDL-C, or total cholesterol levels between the groups (all *p* > 0.05).

The results of the univariate logistic regression analyses evaluating factors associated with CA-AKI are presented in Table 2. Among the demographic and clinical variables, critical RAS (OR: 4.027, 95% CI: 1.576–10.292, *p* = 0.004) and pre-existing CKD (OR: 7.094, 95% CI: 2.639–19.064, *p* < 0.001) were significantly associated with higher odds of CA-AKI. In contrast, age, sex, renal artery lesion location, target renal artery, diabetes mellitus, coronary artery disease, cerebrovascular disease, and antihypertensive treatment regimen were not significantly associated with CA-AKI (all *p* > 0.05).

When evaluated as continuous variables, higher contrast volume (OR: 1.296 per 1-mL increase, 95% CI: 1.163–1.444; *p* < 0.001), glucose (OR: 1.006, 95% CI: 1.002–1.011; *p* = 0.006), triglyceride (OR: 1.004, 95% CI: 1.001–1.008; *p* = 0.027), and uric acid (OR: 1.722, 95% CI: 1.313–2.259; *p* < 0.001) were associated with higher odds of CA-AKI. Higher LVEF (OR: 0.904 per 1% increase, 95% CI: 0.869–0.941; *p* < 0.001), hemoglobin (OR: 0.700, 95% CI: 0.553–0.886; *p* = 0.003), lymphocyte count (OR: 0.145, 95% CI: 0.059–0.359; *p* < 0.001), HDL-C (OR: 0.931, 95% CI: 0.885–0.980; *p* = 0.006), eGFR (OR: 0.954 per 1 mL/min/1.73 m^2^ increase, 95% CI: 0.931–0.977; *p* < 0.001), and albumin (OR: 0.825, 95% CI: 0.733–0.928; *p* = 0.001) were associated with lower odds of CA-AKI.

Among the metabolic indices, the TyG index (OR: 3.005 per 1-unit increase, 95% CI: 1.612–5.599; *p* = 0.001) and AC (OR: 1.437 per 1-unit increase, 95% CI: 1.081–1.910; *p* = 0.013) were significantly associated with CA-AKI, whereas LCI was not significantly associated with CA-AKI when analyzed continuously (OR: 1.022 per 10,000-unit increase, 95% CI: 0.980–1.067; *p* = 0.307).

Spearman rank correlation analysis demonstrated strong positive correlations between triglyceride levels and both the TyG index (ρ = 0.847, *p* < 0.001; n = 147) and LCI (ρ = 0.849, *p* < 0.001; n = 143). The TyG index and LCI were also positively correlated (ρ = 0.666, *p* < 0.001; n = 143). No evidence of problematic multicollinearity was observed in any of the multivariable models (maximum VIF, 1.241; minimum tolerance, 0.806). Box–Tidwell analyses did not identify statistically significant violations of the linearity-in-the-logit assumption for the continuous predictors included in the multivariable models.

In exploratory ROC curve analyses, data-derived cut-off values for CA-AKI were 62.5 mL for contrast volume (AUC = 0.901), 105.5 mg/dL for glucose (AUC = 0.724), 1.08 mg/dL for serum creatinine (AUC = 0.781), 6.75 mg/dL for uric acid (AUC = 0.773), and 9.05 for the TyG index (AUC = 0.732). These thresholds were derived from the same study cohort and were not used to dichotomize variables in the primary logistic regression analyses; they should therefore be considered exploratory and require external validation.

The results of the multivariable logistic regression analyses are presented in Table 3. Three separate models were constructed, each including CKD, LVEF, and contrast volume together with one of the investigated metabolic indices. In Model A, after adjustment for CKD, LVEF, and contrast volume, the TyG index remained significantly associated with CA-AKI (OR: 4.263 per 1-unit increase, 95% CI: 1.120–16.228; *p* = 0.034). Higher LVEF was associated with lower odds of CA-AKI (OR: 0.918 per 1% increase, 95% CI: 0.870–0.969; *p* = 0.002), whereas higher contrast volume was associated with higher odds of CA-AKI (OR: 1.266 per 1-mL increase, 95% CI: 1.093–1.465; *p* = 0.002). CKD did not reach statistical significance in this adjusted model (OR: 4.205, 95% CI: 0.816–21.660; *p* = 0.086).

In Model B, AC was not significantly associated with CA-AKI after adjustment for CKD, LVEF, and contrast volume (OR: 1.581 per 1-unit increase, 95% CI: 0.891–2.807; *p* = 0.118). Similarly, LCI was not significantly associated with CA-AKI in Model C (OR: 1.047 per 10,000-unit increase, 95% CI: 0.966–1.136; *p* = 0.264). LVEF and contrast volume remained significantly associated with CA-AKI in both models.

In sensitivity analyses using Firth penalized logistic regression, the association between the TyG index and CA-AKI remained significant (OR: 3.48 per 1-unit increase, 95% CI: 1.17–14.33; *p* = 0.020). In contrast, neither AC (OR: 1.53 per 1-unit increase, 95% CI: 0.90–2.72; *p* = 0.115) nor LCI (OR: 1.05 per 10,000-unit increase, 95% CI: 0.97–1.12; *p* = 0.212) was significantly associated with CA-AKI. Higher LVEF and higher contrast volume remained significantly associated with CA-AKI in all three penalized models (Supplementary Table S1).

During a median follow-up of 21.97 months (IQR: 13.43–32.57 months), the composite clinical endpoint occurred in 12 patients (8.2%). Patients who developed CA-AKI had a significantly higher incidence of the composite clinical endpoint than those without CA-AKI (27.3% vs. 4.8%, *p* = 0.003). Myocardial infarction was the most frequent event (n = 8, 5.4%), followed by stroke (n = 2, 1.4%), sudden cardiac death (n = 1, 0.7%), and renal artery restenosis requiring repeat revascularization (n = 1, 0.7%).

## 4. Discussion

In the present study, CA-AKI occurred in 15.0% of patients with atherosclerotic RAS undergoing renal angiography, a frequency comparable to that reported in previous studies [13]. More importantly, a higher TyG index remained significantly associated with CA-AKI when evaluated as a continuous variable after adjustment for CKD, LVEF, and contrast volume, and this association was preserved in Firth penalized logistic regression. In contrast, although AC was associated with CA-AKI in univariable analysis and LCI levels were higher among patients who developed CA-AKI, neither AC nor LCI remained significantly associated with CA-AKI in the adjusted continuous-variable analyses.

Consistent with previous studies, pre-existing CKD was strongly associated with CA-AKI in the univariable analysis. However, its association was attenuated and did not reach statistical significance in the revised multivariable models, with wide confidence intervals likely reflecting the limited number of CA-AKI events. Baseline renal dysfunction is a well-established determinant of contrast-associated renal injury because reduced renal functional reserve, impaired autoregulatory capacity, renal medullary hypoxia, and increased oxidative stress render the kidneys more vulnerable to renal injury following contrast exposure [12,14,15]. In contrast, higher LVEF remained consistently associated with lower odds of CA-AKI across the adjusted models and Firth sensitivity analyses [14]. Although the association between left ventricular systolic function and CA-AKI has not been extensively investigated in patients undergoing renal artery angiography, reduced cardiac function may contribute to renal hypoperfusion, diminished oxygen delivery, and prolonged renal exposure to contrast media, thereby increasing susceptibility to renal injury. Collectively, these findings underscore the importance of evaluating both baseline renal function and cardiac performance for preprocedural risk assessment in patients with atherosclerotic RAS undergoing renal angiography.

Among the investigated metabolic biomarkers, the TyG index showed the most consistent association with CA-AKI in patients with atherosclerotic RAS undergoing renal angiography. When analyzed as a continuous variable, each 1-unit increase in the TyG index was associated with approximately four-fold higher adjusted odds of CA-AKI after accounting for CKD, LVEF, and contrast volume (OR: 4.263, 95% CI: 1.120–16.228; *p* = 0.034). Importantly, this association remained significant in the Firth penalized sensitivity analysis (OR: 3.48, 95% CI: 1.17–14.33; *p* = 0.020). This association is biologically plausible because insulin resistance promotes endothelial dysfunction, oxidative stress, chronic low-grade inflammation, and microvascular dysfunction, thereby potentially increasing susceptibility to CA-AKI following contrast exposure [16]. Consistent with our findings, a recent systematic review and meta-analysis demonstrated that an elevated TyG index is associated with an increased risk of acute kidney injury across diverse clinical settings, supporting its role as a marker of renal vulnerability [17]. Our study extends these observations to patients undergoing renal artery angiography, suggesting that the metabolic milieu reflected by the TyG index may identify patients with greater susceptibility to contrast-associated renal injury in this particularly high-risk population. Given its low cost, widespread availability, and ease of calculation from routine laboratory parameters, the TyG index warrants further investigation as a potential adjunct to conventional preprocedural risk assessment.

Among the investigated procedural variables, higher contrast volume remained significantly associated with CA-AKI when analyzed continuously and after adjustment for CKD, LVEF, and each investigated metabolic index. In the TyG model, each 1-mL increase in contrast volume was associated with higher odds of CA-AKI (OR: 1.266, 95% CI: 1.093–1.465; *p* = 0.002), and the association remained significant in the Firth penalized sensitivity analysis. Several mechanisms have been proposed to explain the association between iodinated contrast exposure and renal injury, including tubular toxicity, renal medullary hypoxia, oxidative stress, and intrarenal vasoconstriction. Previous studies have consistently demonstrated that increasing contrast volume is one of the strongest modifiable procedural risk factors for CA-AKI, particularly in patients undergoing complex endovascular or coronary interventions [18,19,20]. However, contrast volume may also serve as a marker of procedural complexity rather than representing an isolated causal exposure. In the present cohort, patients undergoing renal artery intervention received significantly more contrast than those undergoing diagnostic renal angiography alone. Therefore, residual confounding related to procedure type and procedural complexity cannot be excluded, and the magnitude of the observed association between contrast volume and CA-AKI should be interpreted cautiously. Nevertheless, minimizing unnecessary contrast exposure whenever feasible remains a reasonable procedural consideration, particularly in patients at increased risk of CA-AKI.

In contrast to the TyG index, the associations of AC and LCI with CA-AKI were not maintained in the adjusted analyses. AC was significantly associated with CA-AKI in the univariable analysis but not after adjustment for CKD, LVEF, and contrast volume or in the Firth penalized sensitivity analysis. LCI was not significantly associated with CA-AKI when evaluated continuously in either the univariable or multivariable analyses, despite higher median LCI levels among patients who developed CA-AKI. Although no previous study has specifically investigated the relationship between AC, LCI and CA-AKI, growing evidence indicates that lipid metabolism plays an important role in the pathogenesis of contrast-associated renal injury. In this context, previous studies have demonstrated that lipid-related biomarkers, including the ApoB/ApoA-I ratio, are independently associated with the development of CI-AKI following percutaneous coronary intervention, highlighting the potential association between atherogenic dyslipidemia and susceptibility to CA-AKI [21]. Because AC and LCI integrate multiple atherogenic and anti-atherogenic lipid fractions into a single composite index, they may more comprehensively reflect the cumulative metabolic burden, contributing to endothelial dysfunction, oxidative stress, and renal microvascular injury than individual lipid parameters alone. Furthermore, elevated LCI has been associated with adverse cardiovascular outcomes and increased mortality in other high-risk populations, supporting its value as an integrated marker of cardiometabolic risk [10,11]. However, in the present cohort, neither AC nor LCI showed a statistically significant association with CA-AKI after multivariable adjustment.

Notably, both the TyG index and LCI were strongly correlated with triglyceride levels (ρ = 0.847 and ρ = 0.849, respectively), and the two indices were also positively correlated with each other (ρ = 0.666). This finding is expected because triglycerides are incorporated into the calculation of both indices and indicates that they capture partially overlapping metabolic information. Nevertheless, their associations with CA-AKI were not equivalent, as the TyG index remained significantly associated with CA-AKI in the adjusted and Firth penalized analyses whereas LCI did not. These findings should therefore be interpreted as supporting an association of the TyG index with CA-AKI susceptibility rather than demonstrating incremental predictive value over established clinical risk factors.

This study has several limitations. First, its retrospective, single-center design may have introduced selection bias and limits the generalizability of our findings. Second, the relatively small sample size and limited number of CA-AKI events may have reduced the statistical power of the analyses and restricted the number of variables that could be included in the multivariable models. In particular, only 22 CA-AKI events occurred, resulting in wide confidence intervals for some estimates. To address potential small-sample bias, multivariable model complexity was deliberately restricted and Firth penalized logistic regression was performed as a sensitivity analysis; nevertheless, residual model instability and overfitting cannot be completely excluded. Third, all metabolic biomarkers were calculated from a single baseline laboratory measurement obtained before the procedure, and their temporal changes after contrast exposure were not assessed. In addition, the ROC-derived thresholds were identified and evaluated within the same cohort without internal or external validation and should therefore be considered exploratory rather than clinically established cut-off values. In addition, CA-AKI was defined solely on the basis of serum creatinine changes, as urine-output data were not consistently available because of the retrospective study design. Finally, although peri-procedural management was performed according to routine institutional practice, detailed and standardized information regarding hydration protocols, nephroprotective measures, recent contrast exposure, and exposure to potentially nephrotoxic medications was not consistently available because of the retrospective study design and therefore could not be included in the analyses. In addition, because contrast volume was significantly higher among patients undergoing renal artery intervention than among those undergoing diagnostic angiography alone, contrast volume may partly reflect procedural complexity, and residual confounding related to procedure type or procedural characteristics cannot be excluded. Consequently, residual confounding cannot be completely excluded. Furthermore, the secondary clinical endpoint occurred in only 12 patients and should therefore be interpreted as exploratory. Larger prospective multicenter studies are warranted to validate our findings before these biomarkers can be incorporated into routine clinical risk assessment.

## 5. Conclusions

In patients with atherosclerotic renal artery stenosis undergoing renal angiography with or without renal artery intervention, a higher TyG index was associated with increased odds of CA-AKI after adjustment for CKD, LVEF, and contrast volume, and this association remained significant in Firth penalized sensitivity analysis. In contrast, AC and LCI were not significantly associated with CA-AKI in the adjusted continuous-variable analyses. Higher contrast volume and lower LVEF were consistently associated with CA-AKI across the multivariable models. These findings support further investigation of the TyG index as a readily available marker of CA-AKI susceptibility; however, prospective multicenter validation is required before its incorporation into clinical risk assessment.

## Figures and Tables

**Table 1:** Baseline Characteristics of the Study Population According to CA-AKI.

Characteristics	All Patients	No CA-AKI	CA-AKI	*p*
	N = 147	n = 125	n = 22	
Age	65.00 (56.00–72.00)	64.00 (55.00–71.00)	66.50 (61.00–76.50)	0.085
Sex				0.286
- Male	85 (57.8%)	70 (56.0%)	15 (68.2%)	
- Female	62 (42.2%)	55(44.0%)	7 (31.8%)	
RAS				0.002
- Non-critical	101 (68.7%)	92 (73.6%)	9 (40.9%)	
- Critical	46 (31.3%)	33 (26.4%)	13 (59.1%)	
Lesion Location				0.561
- Ostial	142 (96.6%)	121 (96.8%)	21 (95.5%)	
- Distal	5 (3.4%)	4 (3.2%)	1 (4.5%)	
Target Renal Artery				0.399
- Right	50 (34.0%)	43 (34.4%)	7 (31.8%)	
- Left	60 (40.8%)	53 (42.4%)	7 (31.8%)	
- Bilateral	37 (25.2%)	29 (23.2%)	8 (36.4%)	
Procedure Type				0.085
- Renal angiography alone	116 (78.9%)	102 (81.6%)	14 (63.6%)	
- Renal artery intervention	31 (21.1%)	23 (18.4%)	8 (36.4%)	
Comorbidities				
- HTN	145 (98.6%)	123 (98.4%)	22 (100%)	1.000
- DM	43 (29.3%)	33 (26.4%)	10 (45.5%)	0.070
- CAD	117 (79.6%)	97 (77.6%)	20 (90.9%)	0.249
- CeVD	8 (5.4%)	7 (5.6%)	1 (4.5%)	1.000
- CKD	44 (29.9%)	29 (23.2%)	15 (68.2%)	<0.001
Antihypertensive Therapy Regimen				0.427
- Monotherapy	28 (19.0%)	26 (20.8%)	2 (9.1%)	
- Dual Therapy	47 (32.0%)	39 (31.2%)	8 (36.4%)	
- ≥3 Drug Therapy	71 (48.3%)	59 (47.2%)	12 (54.5%)	
Contrast volume (mL)	60.00 (55.00–65.00)	60.00 (55.00–60.00)	70.00 (65.00–86.00)	<0.001
LVEF	60.00 (50.00–60.00)	60.00 (55.00–60.00)	35.00 (30.00–55.00)	<0.001
Hemoglobin	13.10 (11.70–14.30)	13.20 (12.05–14.45)	11.40 (10.20–13.83)	0.003
Neutrophil	5.15 (4.16–6.34)	5.09 (3.99–6.26)	5.67 (4.56–7.50)	0.093
Lymphocyte	2.06 (1.64–2.72)	2.25 (1.71–2.82)	1.52 (0.92–1.82)	<0.001
Monocyte	0.49 (0.38–0.63)	0.49 (0.38–0.63)	0.49 (0.33–0.66)	0.786
Platelet	254.00 (204.00–306.00)	256.00 (212.00–305.00)	216.00 (170.00–320.00)	0.160
Glucose	116.00 (96.00–166.00)	109.00 (94.50–152.50)	155.00 (115.75–214.50)	0.001
HbA1C	5.90 (5.60–6.90)	5.80 (5.60–6.50)	6.80 (5.68–7.75)	0.075
Triglyceride	149.00 (107.00–211.00)	145.00 (94.00–210.00)	199.50 (148.50–257.50)	0.006
LDL-C	115.00 (91.00–143.00)	112.00 (88.00–147.00)	120.00 (105.00–132.75)	0.545
HDL-C	42.00 (35.00–50.00)	43.00 (35.00–50.50)	35.00 (29.00–42.25)	0.005
Total-C	187.00 (162.00–225.00)	187.00 (160.50–225.50)	190.00 (162.00–212.00)	0.937
Creatinine	0.96 (0.79–1.34)	0.92 (0.79–1.23)	1.44 (1.11–1.64)	<0.001
eGFR, mL/min/1.73 m^2^	70.00 (54.00–91.00)	76.00 (57.50–92.50)	42.50 (38.50–64.30)	<0.001
eGFR categories				0.001
≥60	95 (64.6%)	88 (70.4%)	7 (31.8%)	
30–59	47 (32.0%)	34 (27.2%)	13 (59.1%)	
<30	5 (3.4%)	3 (2.4%)	2 (9.1%)	
Uric Acid	5.80 (4.80–7.40)	5.40 (4.50–6.80)	7.65 (6.63–8.33)	<0.001
Albumin	41.00 (39.00–43.60)	41.40 (39.45–44.00)	38.70 (35.90–40.25)	0.001
TyG Index	9.13 (8.71–9.64)	9.02 (8.59–9.55)	9.60 (9.19–10.09)	0.001
AC	3.76 (2.61–4.64)	3.64 (2.46–4.53)	4.32 (3.52–5.51)	0.018
LCI	79,422.00 (36,909.89–167,712.29)	69,696.00 (32,868.77–165,605.83)	111,022.96 (83,447.25–178,419.05)	0.045

Abbreviations: CA-AKI, contrast-associated acute kidney injury; RAS, renal artery stenosis; HTN, hypertension; DM, diabetes mellitus; CAD, coronary artery disease; CeVD, cerebrovascular disease; CKD, chronic kidney disease; mL, milliliter; LVEF, left ventricular ejection fraction; HbA1c, hemoglobin A1c; LDL-C, low-density lipoprotein cholesterol; HDL-C, high-density lipoprotein cholesterol; Total-C, total cholesterol; eGFR, estimated glomerular filtration rate; TyG, triglyceride–glucose; AC, atherogenic coefficient; LCI, lipoprotein combined index.

**Table 2:** Univariate Logistic Regression Analyses of Factors Associated with CA-AKI in Patients with Atherosclerotic RAS.

Factors	Univariate	
	OR (95% CI)	*p*
Age ^1^	1.896 (0.743–4.838)	0.181
Gender ^2^	0.594 (0.226–1.558)	0.290
RAS ^3^	4.027 (1.576–10.292)	**0.004**
RAS location ^4^	1.440 (0.153–13.528)	0.749
Target renal artery ^5^		0.408
Left	0.811 (0.264–2.492)	0.715
Bilateral	1.695 (0.554–5.185)	0.355
DM ^6^	2.323 (0.918–5.880)	0.075
CAD ^7^	2.887 (0.636–13.108)	0.170
CeVD ^8^	0.803 (0.094–6.864)	0.841
CKD ^9^	7.094 (2.639–19.064)	**<0.001**
Antihypertensive Therapy Regimen ^10^		0.451
Dual Therapy	2.667 (0.524–13.570)	0.237
≥3 Drug Therapy	2.644 (0.552–12.664)	0.224
Contrast volume	1.296 (1.163–1.444)	**<0.001**
LVEF	0.904 (0.869–0.941)	**<0.001**
Hemoglobin	0.700 (0.553–0.886)	**0.003**
Neutrophil	1.181 (0.981–1.423)	0.079
Lymphocyte	0.145 (0.059–0.359)	**<0.001**
Monocyte	0.546 (0.042–7.083)	0.643
Platelet	0.996 (0.990–1.002)	0.194
Glucose	1.006 (1.002–1.011)	**0.006**
HbA1C	1.199 (0.981–1.465)	0.076
Triglyceride	1.004 (1.001–1.008)	**0.027**
LDL-C	1.001 (0.989–1.012)	0.923
HDL-C	0.931 (0.885–0.980)	**0.006**
Total-C	0.998 (0.988–1.008)	0.725
Creatinine	1.392 (0.861–2.252)	0.177
Uric Acid	1.722 (1.313–2.259)	**<0.001**
Albumin	0.825 (0.733–0.928)	**0.001**
TyG Index	3.005 (1.612–5.599)	**0.001**
AC	1.437 (1.081–1.910)	**0.013**
LCI	1.022 (0.980–1.067)	0.307

Reference groups: ^1^ <65 years; ^2^ male sex; ^3^ non-critical RAS; ^4^ ostial lesion; ^5^ right renal artery; ^6^ no pre-existing DM; ^7^ no pre-existing CAD; ^8^ no pre-existing CeVD; ^9^ no pre-existing CKD; ^10^ antihypertensive monotherapy. CA-AKI, contrast-associated acute kidney injury; RAS, renal artery stenosis; DM, diabetes mellitus; CAD, coronary artery disease; CeVD, cerebrovascular disease; CKD, chronic kidney disease; LVEF, left ventricular ejection fraction; HbA1c, hemoglobin A1c; LDL-C, low-density lipoprotein cholesterol; HDL-C, high-density lipoprotein cholesterol; Total-C, total cholesterol; TyG, triglyceride–glucose; AC, atherogenic coefficient; LCI, lipoprotein combined index. Bold values indicate statistical significance (*p* < 0.05). Except for age, which was analyzed categorically (<65 vs. ≥65 years), continuous variables were entered into the univariable logistic regression analyses in their continuous form. Odds ratios represent the change in the odds of CA-AKI per one-unit increase in each continuous variable, except for LCI, which was rescaled and expressed per 10,000-unit increase to improve interpretability.

**Table 3:** Multivariable Logistic Regression Analysis of Factors Associated with CA-AKI in Patients with Atherosclerotic RAS.

Factors	OR (95% CI)	*p*
Model A		
CKD ^1^	4.205 (0.816–21.660)	0.086
LVEF per 1% increase	0.918 (0.870–0.969)	**0.002**
TyG Index per 1-unit increase	4.263 (1.120–16.228)	**0.034**
Contrast volume, per 1 mL increase	1.266 (1.093–1.465)	**0.002**
Model B		
CKD ^1^	2.338 (0.516–10.604)	0.271
LVEF per 1% increase	0.916 (0.871–0.964)	**0.001**
AC, per 1-unit increase	1.581 (0.891–2.807)	0.118
Contrast volume per 1 mL increase	1.275 (1.111–1.462)	**0.001**
Model C		
CKD ^1^	2.652 (0.583–12.058)	0.207
LVEF per 1% increase	0.915 (0.869–0.964)	**0.001**
LCI per 10,000-unit increase	1.047 (0.966–1.136)	0.264
Contrast volume, per 1 mL increase	1.264 (1.103–1.447)	**0.001**

Reference group for the categorical variable: ^1^ no pre-existing CKD. Continuous variables were entered into the multivariable logistic regression models in their continuous form. Odds ratios represent the change in the odds of CA-AKI per one-unit increase in each continuous variable, except for LCI, which was rescaled and expressed per 10,000-unit increase to improve interpretability. Model A included CKD, LVEF, contrast volume, and TyG index. Model B included CKD, LVEF, contrast volume, and AC. Model C included CKD, LVEF, contrast volume, and LCI. Abbreviations: CA-AKI, contrast-associated acute kidney injury; RAS, renal artery stenosis; CKD, chronic kidney disease; LVEF, left ventricular ejection fraction; TyG, triglyceride–glucose; AC, atherogenic coefficient; LCI, lipoprotein combined index. Bold values indicate statistical significance (*p* < 0.05).

## Data Availability

The data that support the findings of this study are not publicly available due to patient privacy and institutional restrictions but are available from the corresponding author upon reasonable request.

## Supplementary Materials

The following supporting information can be downloaded at: https://www.mdpi.com/article/10.3390/jcdd13090447/s1, Figure S1: Study Flow Diagram; Table S1: Firth Penalized Logistic Regression Sensitivity Analyses for Factors Associated with CA-AKI.
