# Supplementary material for: Association of the Triglyceride–Glucose Index with Contrast-Associated Acute Kidney Injury in Patients with Atherosclerotic Renal Artery Stenosis
Source: J Cardiovasc Dev Dis. 2026 Sep 8;13(9):447. doi: 10.3390/jcdd13090447 (PMC13607574; doi:10.3390/jcdd13090447)
Supplement: Supplementary file 1 [file jcdd-13-00447-s001.zip › jcdd-4527130-supplementary.pdf]

Figure S1. Study Flow Diagram

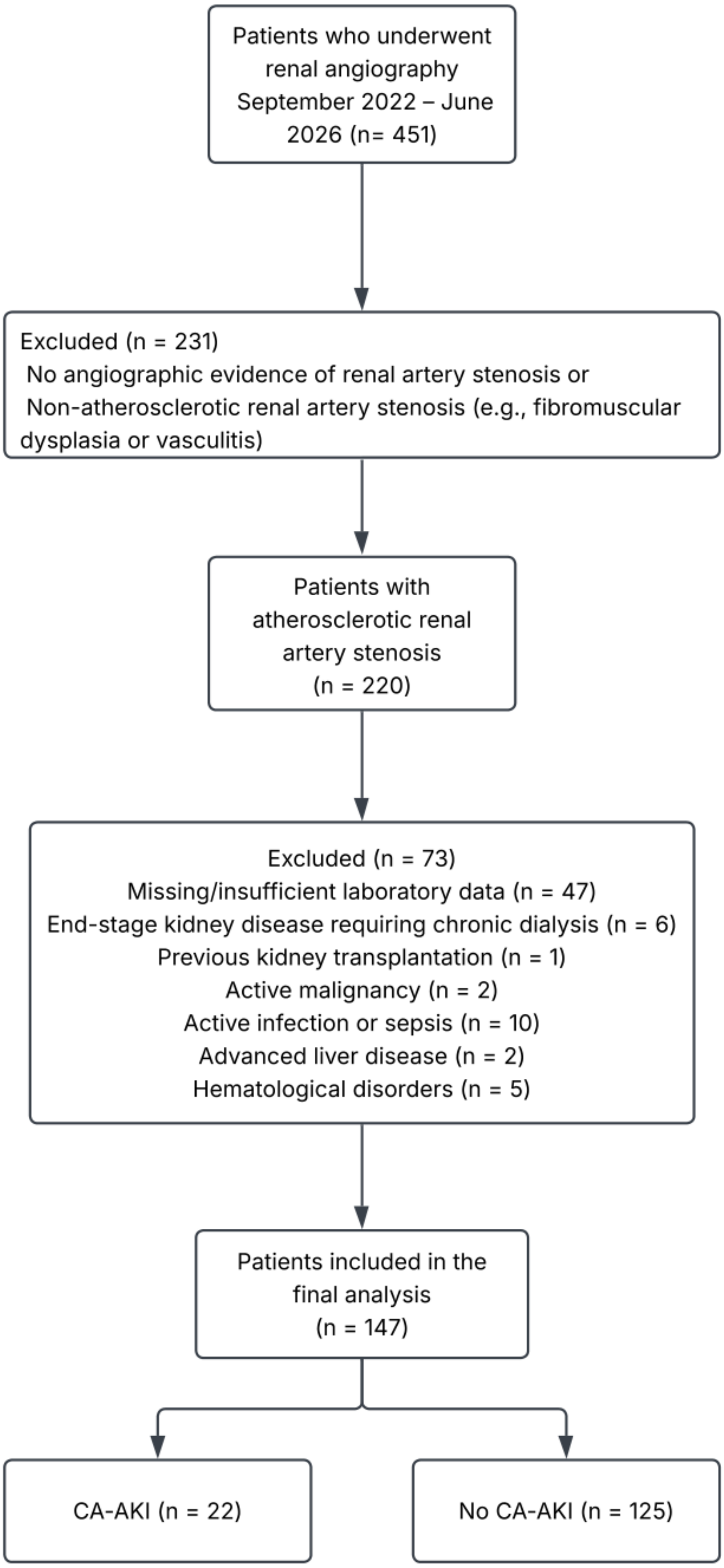

Supplementary Table S1. Firth Penalized Logistic Regression Sensitivity Analyses for Factors Associated with CA-AKI

Factor	Penalized OR (95% CI)	p
**Model A- TyG (n=147)**		
**CKD**	3.64 (0.84-18.23)	0.080
**LVEF, per 1% increase**	**0.93 (0.88-0.97)**	**0.001**
**TyG index, per 1-unit increase**	**3.48 (1.17-14.33)**	**0.020**
**Contrast volume, per 1 mL increase**	**1.21 (1.10-1.43)**	**<0.001**
**Model B- AC (n=147)**		
**CKD**	2.19 (0.53-9.26)	0.231
**LVEF, per 1% increase**	**0.93 (0.88-0.97)**	**<0.001**
**AC, per 1-unit increase**	1.53 (0.90-2.72)	0.115
**Contrast volume, per 1 mL increase**	**1.22 (1.11-1.43)**	**<0.001**
**Model C- LCI (n=143)**		
**CKD**	2.37 (0.58-10.01)	0.216
**LVEF, per 1% increase**	**0.92 (0.88-0.97)**	**<0.001**
**LCI, per 10,000-unit increase**	1.05 (0.97-1.12)	0.212
**Contrast volume, per 1 mL increase**	**1.21 (1.11-1.41)**	**<0.001**

Model A included CKD, LVEF, contrast volume, and TyG index. Model B included CKD, LVEF, contrast volume, and AC. Model C included CKD, LVEF, contrast volume, and LCI. Firth penalized logistic regression was performed as a sensitivity analysis to reduce small-sample bias related to the limited number of CA-AKI events. Continuous variables were entered in their continuous form. Odds ratios represent the change in the odds of CA-AKI per one-unit increase, except for LCI, which was expressed per 10,000-unit increase. Models A and B included 147 patients. Model C was based on 143 complete cases with available LCI data.

Abbreviations: CA-AKI, contrast-associated acute kidney injury; CKD, chronic kidney disease; LVEF, left ventricular ejection fraction; TyG, triglyceride-glucose index; AC, atherogenic coefficient; LCI, lipoprotein combined index.

Bold values indicate statistical significance ($p < 0.05$).
